# Preemptive parasternal intercostal nerve block for patients undergoing off-pump coronary artery bypass grafting: a double-blind, randomized, controlled trial

**DOI:** 10.3389/fcvm.2023.1188518

**Published:** 2023-05-18

**Authors:** Mengmeng Zou, Wei Ruan, Jintao Liu, Junmei Xu

**Affiliations:** ^1^ Center for Rehabilitation Medicine, Department of Anesthesiology, Zhejiang Provincial People's Hospital, Affiliated People's Hospital, Hangzhou Medical College, Hangzhou, China; ^2^ Department of Anesthesiology, The Second XiangYa Hospital of Central South University, Hunan, China

**Keywords:** preemptive parasternal intercostal nerve block, analgesia, hemodynamics, enhanced recovery, off-pump coronary artery bypass grafting

## Abstract

**Background:**

Parasternal intercostal nerve block has been increasingly used for postoperative analgesia and has shown that this technique can provide effective postoperative analgesia. This study aimed to investigate the effect of preemptive parasternal intercostal nerve block on the opioid and vasoactive drug dose required for intraoperative hemodynamic stability and postoperative analgesia in patients undergoing off-pump coronary artery bypass grafting.

**Methods:**

In this prospective, randomized controlled study, 64 participants aged 45–75 years scheduled for off-pump coronary artery bypass grafting at The Second Xiangya Hospital of Central South University. Patients were randomized into two groups and preoperatively administered ropivacaine (group R) and saline (group S), in the parasternal intercostal spaces with ultrasound-guided bilateral nerve block.

**Results:**

The primary outcome was intraoperative sufentanil and vasopressor dosage. The secondary outcomes were intraoperative hemodynamics, postoperative pain scores, and anesthesia recovery, postoperative use of rescue dezocine, stay in intensive care unit, and length of hospital stay. The consumption of intraoperative sufentanil and vasopressor was significantly lower in group R than in group S. The visual analog score in group R was significantly lower than that in group S up to 12 h postoperatively. The time to anesthesia recovery was significantly less in group R than in group S. Most patients in group S required rescue dezocine, whereas most patients in group R did not. The hemodynamic variables were stable in all patients.

**Conclusions:**

A preemptive parasternal intercostal nerve block effectively reduced the required intraoperative sufentanil and norepinephrine dose and provided adequate analgesia for the first 12 h after surgery. Therefore, a preemptive parasternal intercostal nerve block is a good option for patients undergoing off-pump coronary artery bypass grafting.

**Clinical trial registration:**

chictr.org.cn, identifier ChiCTR1800017210.

## 1. Introduction

Off-pump coronary artery bypass grafting (OPCABG) is a common surgical treatment for coronary heart diseases. Intraoperative hemodynamic stability and myocardial protection have been a hot topic in cardiac anesthesiology. Some operations of cardiac surgery, especially sternotomy including skin and subcutaneous tissue incision, sternum splitting and sternal retractor setup, as well as cannulation, may induce an increase in blood pressure and/or heart rate (HR) (1). These episodes are associated with an increase in myocardial oxygen consumption and possible myocardial ischemia. Usually, they are controlled by the administration of a high dose of anesthetic agents (2).

Adverse perioperative pain stimulates the neuroendocrine system and causes stress, which adversely affects the cardiovascular, respiratory, and digestive systems (3, 4). Effective postoperative pain control may be beneficial for early extubation, cost reduction, and rapid recovery (5–7). In addition, appropriate analgesia can reduce pain-related morbidity.

Recently, regional nerve block techniques have increasingly been used for analgesia during surgery. Epidural anesthesia and thoracic paravertebral nerve block provide effective analgesia after cardiac surgery and reduce postoperative mortality (6, 8–12). However, these techniques remain controversial owing to the risk of hematoma caused by inappropriate needle depth especially during antiplatelet or anticoagulant therapy, which is usually administered to elderly patients undergoing OPCABG (13, 14). Peripheral techniques offer safety and efficiency. The parasternal intercostals nerve block primarily anesthetizes the intercostal nerves close to the sternal border and anterior cutaneous branch of the intercostal nerve and is used as an adjuvant for pain management post-cardiac surgery. This modality is highly effective in patients experiencing sternal wound pain following cardiac surgery (15–19). However, preemptive parasternal intercostal nerve block in perioperative benefits are not well established. This study aimed to investigate the effect of preemptive parasternal nerve block on the opioid and vasoactive drug dose required for intraoperative hemodynamic stability and on postoperative analgesia in patients undergoing OPCABG.

## 2. Materials and methods

### 2.1. Study population

This randomized controlled trial was approved by the institutional ethics committee (The Second Xiangya Hospital of Central South University, Chairperson Jing Ping Zhao, ref: 2018-024) on March 29, 2018, and registered with ChiCTR (ref: ChiCTR1800017210). This study was conducted at the Second Xiangya Hospital between July 20, 2018, and September 30, 2018. All patients provided written informed consent before participating in this study. Patient data and postoperative evaluations were double-blinded in this prospective, single-center, randomized, placebo-controlled study. Coronary angiography revealed multivessel coronary artery diseases, and 64 patients planned to undergo OPCABG surgery were selected, regardless of sex. The inclusion criteria were as follows: age, 45–75 years; weight, 40–90 kg; American Society of Anaesthesiologists class III or IV status; and absence of intra-aortic balloon pump, and neuropsychiatric disorders included all patients with chronic pain or patients who take any type of pain medications.

The exclusion criteria were as follows: infection at the block site, left ventricular ejection fraction <50%, chronic liver or kidney disease, allergy to amide-type anesthetics, and low cardiac output syndrome with inotrope and/or intra-aortic balloon pump support. Other reasons for exclusion were as follows: change of treatment plan to cardiopulmonary bypass during the operation, development of postoperative complications requiring reoperation, requirement of intra-aortic balloon pump support intraoperatively or postoperatively, requirement of postoperative reintubation, and sedation for more than 48 h.

### 2.2. Study protocol

After obtaining written informed consent from the participants using the sealed envelope method, they were randomized into two groups: the participants were administered either ropivacaine (group R) or saline (group S). Medication administration and data collection were performed in a double-blinded manner: one anesthesiologist prepared the ropivacaine or saline and administered the block, another anesthesiologist administered anesthesia and collected the data.

**Figure 1:**
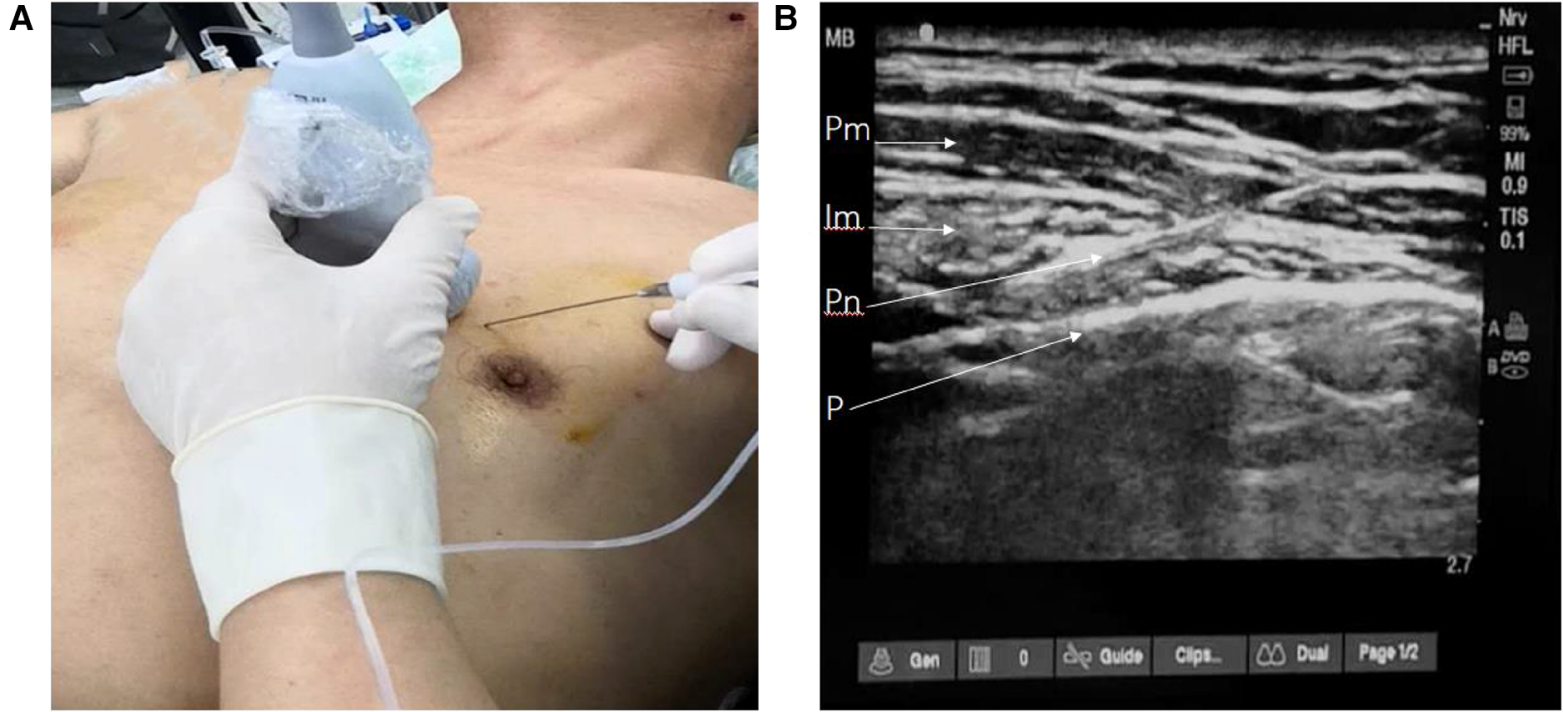
Parasternal intercostal nerve block procedure. (**A**) The needle and probe position, (**B**) ultrasonographic image of parasternal space. Pm, pectoralis major; Im,intercostal muscle; Pn, puncture needle; P, pleura.

For patients receiving the parasternal intercostal nerve block, 0.35% ropivacaine and 1 mg dexamethasone or 0.9% saline were administered in 20 ml aliquots injected into the space between the internal intercostal and the transvs. thoracis muscles on each side, 2 cm–2.5 cm lateral to the sternal edge, using ultrasound guidance (Figure 1). The block was administered by an anesthesiologist in a standardized manner before anesthesia induction.

A standardized anesthesia program was applied for all participants. Anesthesia was induced with midazolam (0.05–0.1 mg kg^–1^), sufentanil (0.3–0.7 ug kg^–1^), vecuronium (0.1–0.2 mg kg^–1^), and etomidate (0.03 mg kg^–1^) and maintained with a target controlled infusion of propofol (1.0–3.0 ugml^–1^) and remifentanil (1.0–4.0 ngml^–1^) and an infusion of cisatracurium (0.1 mg kg^–1^ h^–1^). Electrocardiography, oxygen saturation, HR, central venous pressure, bispectral index (BIS), and arterial blood pressure were monitored intraoperatively. The BIS was maintained between 40 and 55. The basal HR and systolic blood pressure (SBP) were the average values measured in the ward 3 days before surgery. Hypertension and/or tachycardia (the HR and SBP were >20% of the baseline value) were first treated by incremental increases in remifentanil, while the propofol dose was increased only if BIS >55. In these cases, the target concentrations of propofol and remifentanil were increased in steps of 1 ug ml^–1^ and 1 ng ml^–1^, respectively, every 15–20 s, until the appropriate level of anesthesia was achieved. If the effect-site concentration of remifentanil was up to 4.0 ng ml^–1^, sufentanil was added with a single intravenous push of 0.5 ug kg^–1^. Norepinephrine or fluid therapy was provided to treat hypotension [mean arterial pressure (MAP) <65 mmHg] and anisodamine to manage bradycardia (HR <45 bpm).

All patients were transferred to the ICU after surgery and underwent postoperative management. The postoperative analgesia protocol involved a single intravenous push of 5 mg dezocine as required.

The primary outcomes include consumption of sufentanil and intraoperative norepinephrine, intraoperative hemodynamics, pain scores for 48 h after surgery, and postoperative analgesia requirements, the time of anesthetic recovery, ICU stay, and length of hospital stay. The visual analog scale (VAS) was used to assess postoperative pain.

### 2.3. Statistical analyses

Descriptive statistics were used for all study variables, and a two-tailed Student’s *t*-test was performed to assess normally distributed data. Continuous variables are presented as means with standard deviations, and categorical variables are presented as the number of patients in each category and the corresponding percentages. Quantitative variables were compared using the Student’s *t*-test or Mann Whitney *U*-test according to the normality of distribution. Categorical variables were compared using the *χ*^2^ test. The intragroup comparison was performed using repeated measures analysis of variance. The tests were performed using the Statistical Analysis System software. Statistical significance was set to a *P*-value of <0.05.

## 3. Results

**Figure 2:**
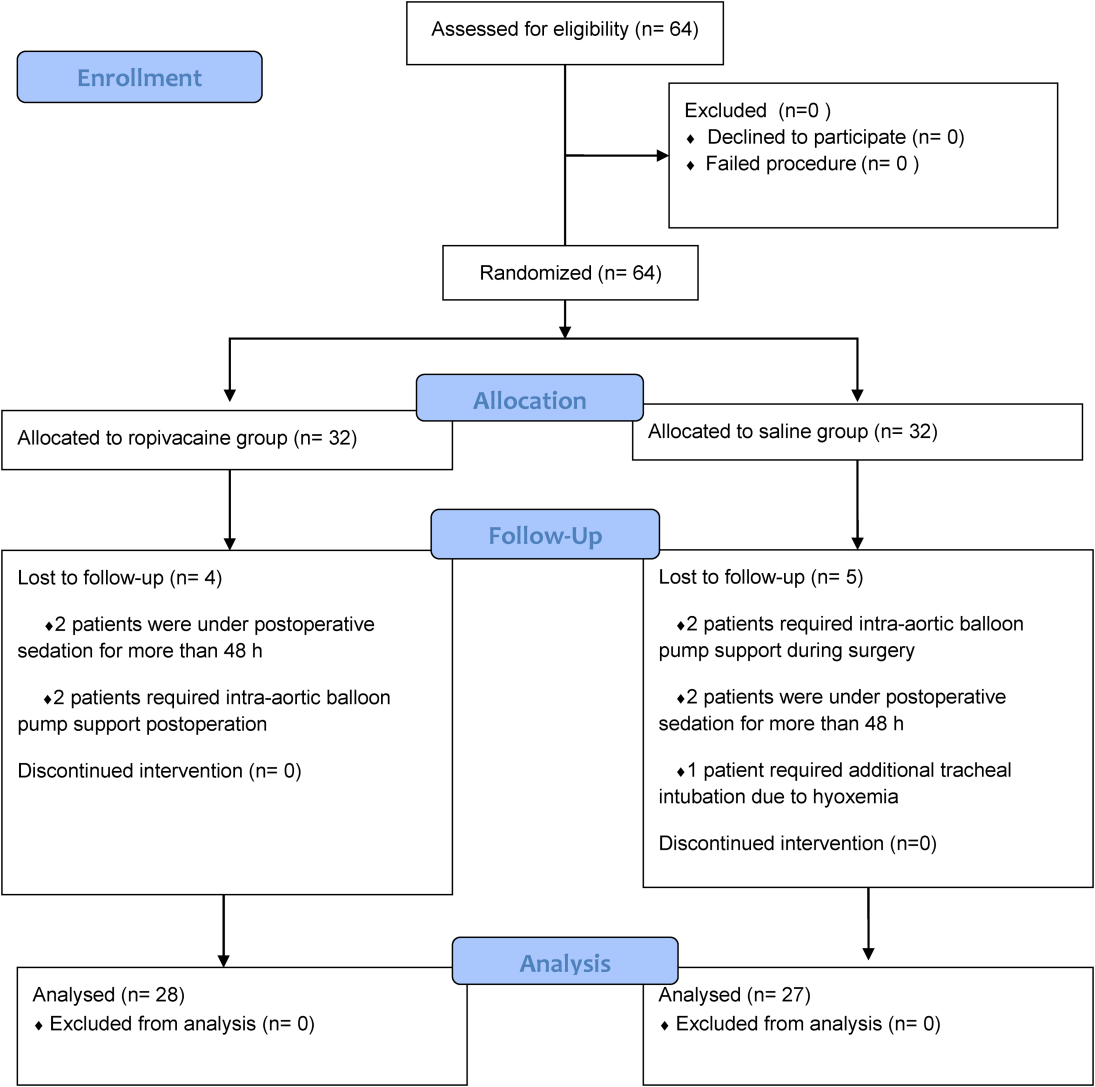
CONSORT Flow diagram.

Sixty-four patients were randomized into group R and group S. However, four patients from group R and five from group S were excluded, leaving 28 patients in group R and 27 in group S (Figure 2). In group R, patients were excluded for the following reasons: two patients were under postoperative sedation for more than 48 h, and two patients required intra-aortic balloon pump support. Of the patients excluded from group S, two required intra-aortic balloon pump support, two were under postoperative sedation for more than 48 h, and one required additional tracheal intubation due to low oxygen in the blood. Patient demographics and baseline clinical characteristics were similar between the two groups (Table 1). Compared to patients who received saline, patients who received ropivacaine required significantly lower doses of sufentanil at the time of skin incision and especially at median sternotomy (Table 2). The total consumption of sufentanil was approximately 50% lower in the ropivacaine group than in the saline group (*P *< 0.001).

**Table 1:** Preoperative patient characteristics.

	Ropivacaine (*n *= 28)	Saline (*n *= 27)	*P* value
Age (year), mean ± SD	59.85 ± 8.53	59.29 ± 8.85	0.812
BMI (kg/m^2^), mean ± SD	24.34 ± 2.49	24.28 ± 2.40	0.918
Sex			
(Male/female)	19/9	13/14	*P* > 0.05
LVEF (%), mean ± SD	65.50 ± 5.03	64.88 ± 6.95	0.710

The baseline demographics of the two groups were comparable. A *P* value <0.05 was considered significant.

BMI, bodymass index; LVEF, left ventricular ejection fraction.

**Table 2:** Intraoperative data.

	Ropivacaine (*n *= 28)	Saline (*n *= 27)	*P* value
Dose of Sufentanil (ug)			
Skin incision	41.60 ± 11.94	68.88 ± 21.45	<0.01
Median sternotomy	45.17 ± 12.21	96.67 ± 21.45	<0.01
Total dose	84.82 ± 25.07	185.37 ± 59.14	<0.01
Remifentanil (ug kg^−1^ h^−1^)	3.81 ± 1.26	3.37 ± 1.40	0.285
Vasoactive drugs			
Maximum dose of norepinephrine (mlh^−1^)	6.34 ± 2.18	8.37 ± 4.03	0.027
Dose of norepinephrine (ml h^−1^)			
T1	0	0	1
T2	1.86 ± 3.03	1.67 ± 2.65	0.805
T3	0	0	1
T4	0.39 ± 0.88	2.11 ± 2.24	<0.01
T5	4.75 ± 2.49	7.63 ± 3.59	0.001
T6	1.54 ± 1.29	3.63 ± 1.92	<0.01
T7	0.50 ± 0.96	1.89 ± 1.92	0.002
Duration of surgery (h)	4.38 ± 1.00	4.78 ± 1.03	0.156

Concentration of norepinephrine was 40 ugml^−1^.

T2, 1 min after skin incision; T3, 1 min after median sternotomy; T4, after dissection of internal mammary artery; T5, 5 min after reperfusion; T6, closure of sternum; T7, at the end of the surgery.

**Figure 3:**
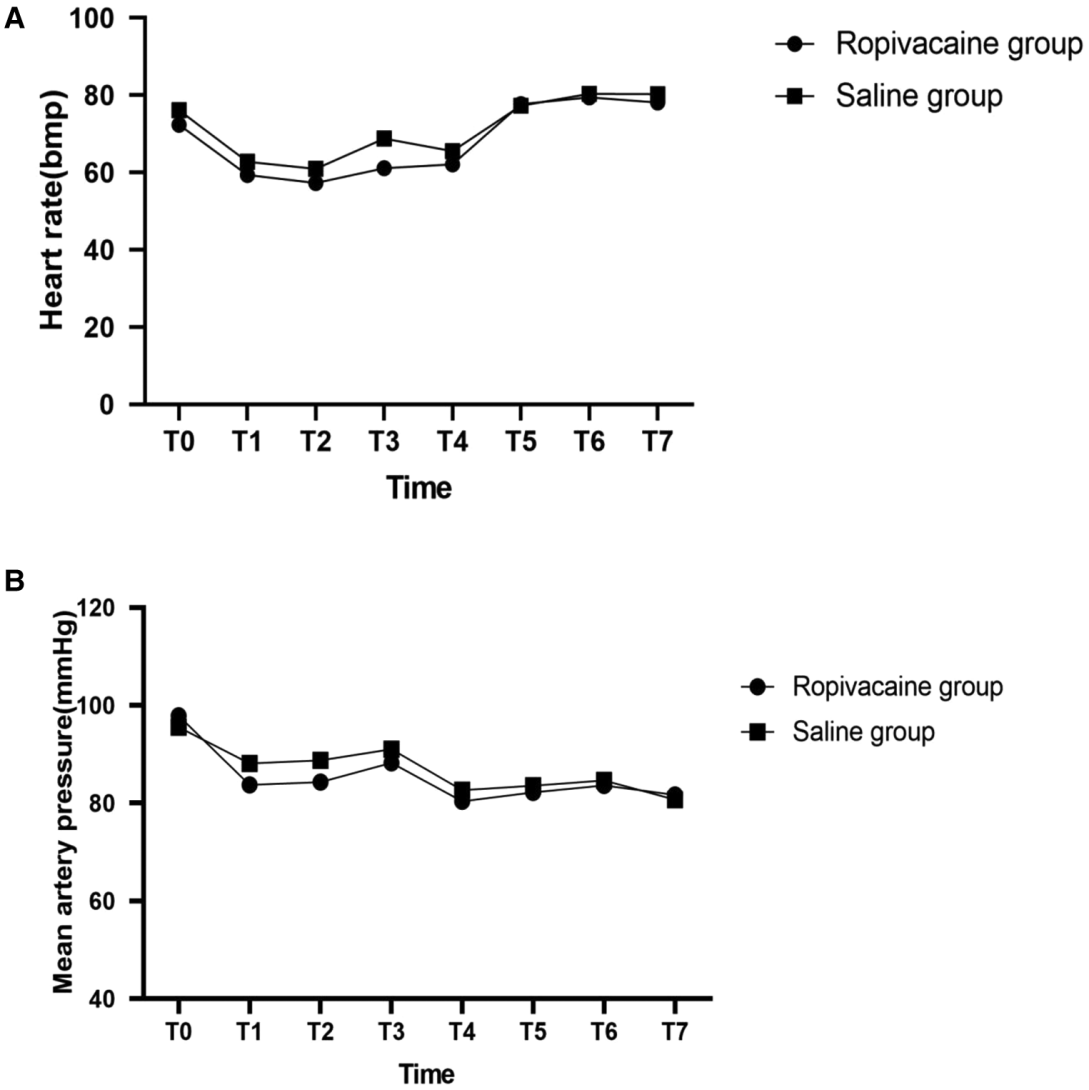
Heartrate (**A**) and mean artery pressure (**B**) with time in the two groups. T0, entering operation room; T1, before skin incision; T2, 1 min after skin incision; T3, 1 min after median sternotomy; T4, after dissection of internal mammary artery; T5, 5 min after reperfusion; T6, closure of sternum; T7, at the end of the surgery.

Hemodynamic data showed that the patients in the two groups were hemodynamically stable. Hemodynamics (HR and MAP) were not significantly different between both groups (Figure 3). However, at the time of dissection of the internal mammary artery, 5 min after reperfusion, closure of the sternum, and the end of the surgery, patients in group R required a significantly lower dose of norepinephrine than those in group S (*P *< 0.05) (Table 2).

**Figure 4:**
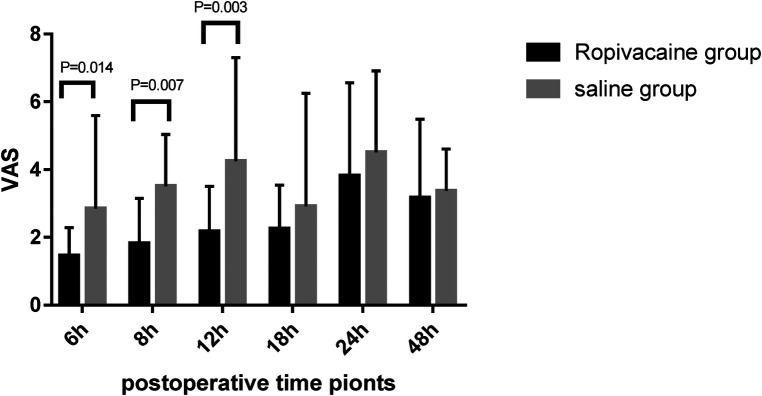
Visual analog scale scores for pain in the first 48 h after surgery (mean ± standard deviation).

The pain scores (VAS) over a 48 h period are shown in Figure 4. Patients in the ropivacaine group experienced less pain during the first 12 h after surgery than those in the saline group. The VAS scores of the ropivacaine group were lower than those of the saline group 6, 8, and 12 h after the operation (*P *< 0.05). The difference in the VAS scores of the two groups at 18, 24, and 48 h after the operation was not statistically significant. Most patients in the ropivacaine group did not require rescue dezocine, whereas most patients in the saline group did (*P *< 0.05) (Table 3). The time to recovery from anesthesia was significantly lower in the ropivacaine group than in the saline group (*P *< 0.05). Nevertheless, no effect was observed on the length of stay in the ICU or hospital after surgery in the ropivacaine group (Table 3).

**Table 3:** Postoperative data.

	Ropivacain (*n *= 28)	Saline (*n *= 27)	*P* value
Anaesthetic recovery time (h)	1.7 ± 1.3	2.8 ± 2.0	0.023
ICU stay (h)	33.3 ± 15.7	35.9 ± 20.9	0.609
The length of hospital stay after surgery (days)	10.6 ± 4.1	10.4 ± 2.5	0.828
Analgesic dose required (%)			
None	17 (60.7)	9 (33.3)	0.042
Dezocine			
10 mg	3 (10.7)	10 (37.0)	
>10 mg	8 (28.6)	8 (29.6)	

Data are presented as mean ± standard deviation or *n* (%).

## 4. Discussion

Hemodynamic stability is a significant concern in the intraoperative management of OPCABG. In the past, large doses of opioids were used in thoracotomy to inhibit the stress response caused by strong stimuli, such as skin incision, saw sternum, and chest closure (2). However, opioids are associated with side effects, such as inhibition of the cardiovascular system, making them unsuitable for maintenance of intraoperative hemodynamics. A preemptive parasternal intercostal nerve block could provide an effective level of anesthesia of the chest wall. Our study demonstrated that ropivacaine administration through a parasternal intercostal nerve block before anesthesia induction, helped decrease the required intraoperative opioid dose. There was no difference in the hemodynamic parameters between the ropivacaine and saline groups, but norepinephrine consumption was decreased in the ropivacaine group, this may owing to decreasing narcotic doses may contribute to more hemodynamic stability during the less stimulating portion of the surgery, which probably indicates that a parasternal nerve block can help maintain hemodynamic stability to a certain extent.

In recent years, combining general anesthesia with regional anesthesia has become a popular topic. Saad et al.showed that preemptive thoracic paravertebral and serratus anterior plane block provide comparable levels of adequate analgesia for the first 24 h after thoracotomy, with easier application and lower complication rates. The two procedures reduce intraoperative fentanyl and postoperative morphine consumption (20). In our study, we used the technique to compare ultrasound-guided parasternal intercostal nerve blocks with ropivacaine and saline. Local anesthetics can be injected into the internal intercostal muscle. The results of this study indicate the analgesic efficacy of the parasternal intercostal nerve block. Further, the parasternal intercostal nerve block is safer than the thoracic paravertebral block because of its lower rate of adverse events, especially hypotension and bradycardia (21).

Most studies on parasternal intercostal nerve block for postoperative analgesia have been conducted in patients requiring median sternotomy. Chen et al. studied the postoperative analgesia obtained with an ultrasound-guided parasternal intercostals nerve block in patients undergoing mediastinal mass resection by median sternotomy. They found that the parasternal intercostal nerve block effectively reduced postoperative pain and adjuvant analgesic requirement (22). Padaa et al.compared the effects of preincisional and postincisional parasternal intercostal blocks on postoperative pain in cardiac surgery and found that the intraoperative fentanyl requirement before cardiopulmonary bypass was significantly reduced and that both blocks provided comparable pain relief postoperatively (23). Abadi et al. evaluated the effectiveness of the parasternal intercostal nerve block in patients undergoing coronary artery bypass grafting with sternotomy as part of the enhanced recovery after surgery (ERAS) pain management protocol as compared with that in a non-ERAS patient group. They observed lower maximum pain scores and the requirement of a lower opioid dose for postoperative analgesia on administering the block. The data from our study also indicate that the parasternal intercostal block provided good postoperative analgesia. In addition, our study showed that the parasternal intercostal block reduced intraoperative opioid and norepinephrin use, which indirectly indicates that a preemptive parasternal intercostal block may help maintain intraoperative hemodynamic stability in patients with OPCABG.

The present study demonstrated that the parasternal intercostal nerve block provided adequate analgesia for the first 12 h after surgery. Pain intensity was significantly lower in the ropivacaine group than in the saline group after 12 h. One or more doses of dezocine were required during the first 48 h postoperatively in all patients, except in 17 patients in the ropivacaine group. The saline group required higher doses of dezocine for all except nine patients. Therefore, although a parasternal intercostal nerve block reduced dezocine consumption, it was not adequate as the only postoperative analgesic. In the current study, the effect of the local anesthetic in the ropivacaine group lasted up to 12 h after surgery. This may be related to the inclusion of dexamethasone to enhance the compatibility with local anesthetics. Studies have shown that local anesthetics with dexamethasone can effectively extend the action time of local anesthetics (24, 25).

In this study, we adopted the concept of preemptive analgesia through the application of regional blocks before surgical incisions. This approach focuses on postoperative analgesia in addition to the prevention of central sensitization and chronic neuropathic pain (26).

Poorly managed pain following cardiac surgery can lead to an increased risk of complications, such as lung collapse and chest infections, due to altered mechanical functions of the lungs and ventilation–perfusion mismatch. Pascarella et al. showed that parasternal block provided a better postoperative performance at spirometry compared to the control group (27). Effective relief of sternal pain is important for both patients and physicians. Increasing evidence suggests that severe pain in the sternum after surgery significantly affects the time of tracheal tube removal, hemodynamic stability, and subsequent postoperative recovery (4, 28, 29). Barr et al. and McDonald et al. found that a parasternal intercostal block significantly improves blood oxygen levels (16, 17). Our study did not monitor this parameter.

In this study, the time of recovery from anesthesia was lower in the ropivacaine group than in the saline group, which may be related to the lower intraoperative sufentanil dose. However, there was no difference in the ICU monitoring time and postoperative hospital stay between the groups, which may be attributable to strict criteria for ICU transfer. Apart from the parasternal nerve block, recovery of myocardial function, use of postoperative vasoactive drugs, and hemodynamic stability are also important factors that influence the duration of ICU stay.

## 5. Limitations

The outcomes of this study arelimited to the short-term effects of parasternal intercostal nerve block. Hence, its long-term effects require further study. In addition, we did not evaluate intraoperative and postoperative serological indicators, postoperative hemodynamic indicators, postoperative vasoactive drug use, and changes in myocardial enzymes. These aspects should therefore be explored in future studies.

## 6. Conclusion

This study demonstrates an effective and practical treatment for OPCABG. The parasternal intercostal block can help reduce opioid and vasopressors consumption during OPCABG and provide satisfactory analgesia in the earliest hours following surgery.

## Data Availability

The raw data supporting the conclusions of this article will be made available by the authors, without undue reservation.

## Author's note

This article was previously posted to research square. DOI: https://doi.org/10.21203/rs.2.1847/v1 is the submitted manuscript currently on a pre-print server.
